# Infection intensity-dependent accuracy of reagent strip for the diagnosis of *Schistosoma haematobium* and estimation of treatment prevalence thresholds

**DOI:** 10.1371/journal.pntd.0010332

**Published:** 2022-04-25

**Authors:** Carla M. Grolimund, Oliver Bärenbold, Christoph F. Hatz, Birgitte J. Vennervald, Charles Mayombana, Hassan Mshinda, Jürg Utzinger, Penelope Vounatsou

**Affiliations:** 1 Swiss Tropical and Public Health Institute, Allschwil, Switzerland; 2 University of Basel, Basel, Switzerland; 3 Department of Veterinary and Animal Science, University of Copenhagen, Copenhagen, Denmark; 4 Ifakara Health Institute, Dar es Salaam, Tanzania; Centers for Disease Control and Prevention, UNITED STATES

## Abstract

**Background:**

Reagent strip to detect microhematuria as a proxy for *Schistosoma haematobium* infections has been considered an alternative to urine filtration for individual diagnosis and community-based estimates of treatment needs for preventive chemotherapy. However, the diagnostic accuracy of reagent strip needs further investigation, particularly at low infection intensity levels.

**Methods:**

We used existing data from a study conducted in Tanzania that employed urine filtration and reagent strip testing for *S. haematobium* in two villages, including a baseline and six follow-up surveys after praziquantel treatment representing a wide range of infection prevalence. We developed a Bayesian model linking individual *S. haematobium* egg count data based on urine filtration to reagent strip binary test results available on multiple days and estimated the relation between infection intensity and sensitivity of reagent strip. Furthermore, we simulated data from 3,000 hypothetical populations with varying mean infection intensity to infer on the relation between prevalence observed by urine filtration and the interpretation of reagent strip readings.

**Principal findings:**

Reagent strip showed excellent sensitivity even for single measurement reaching 100% at around 15 eggs of *S. haematobium* per 10 ml of urine when traces on reagent strip were considered positive. The corresponding specificity was 97%. When traces were considered negative, the diagnostic accuracy of the reagent strip was equivalent to urine filtration data obtained on a single day. A 10% and 50% urine filtration prevalence based on a single day sampling corresponds to 11.2% and 48.6% prevalence by reagent strip, respectively, when traces were considered negative, and 17.6% and 57.7%, respectively, when traces were considered positive.

**Conclusions/Significance:**

Trace results should be included in reagent strip readings when high sensitivity is required, but excluded when high specificity is needed. The observed prevalence of reagent strip results, when traces are considered negative, is a good proxy for prevalence estimates of *S. haematobium* infection by urine filtration on a single day.

## Author summary

Control of schistosomiasis, a parasitic worm infection affecting more than 200 million people worldwide, relies mainly on mass drug administration of praziquantel to school-age children as well as adults in areas where the disease is particularly rampant. The World Health Organization has set thresholds of observed prevalence that require intervention to reach the goal of eliminating schistosomiasis as a public health problem by 2025. Intervention thresholds are defined based on parasitologic methods, which is urine filtration for *Schistosoma haematobium*. There are alternative diagnostic methods to detect *S. haematobium*, such as the detection of blood in urine that is a common symptom of urogenital schistosomiasis. We determined the diagnostic sensitivity and specificity of a reagent strip to detect microhematuria using data from two villages in Tanzania at multiple time points (once before and several times after treatment with the deworming drug praziquantel) and translate the urine filtration intervention thresholds to reagent strip equivalents. We show that the reagent strip including trace results is almost perfectly sensitive for infections above 15 eggs of *S. haematobium* per 10 ml of urine and that a 10% observed prevalence by urine filtration corresponds to 17.6% observed prevalence by reagent strip including traces.

## Introduction

*Schistosoma haematobium* is the most prevalent of the schistosome species parasitizing humans, causing urogenital schistosomiasis if left untreated [1, 2]. More than 200 million individuals are infected with any species of *Schistosoma* that, collectively, cause a global burden of 2.1 million disability-adjusted life years (DALYs) [3–5]. While mortality is relatively low, there is considerable morbidity that might be manifested as anemia, growth stunting, impaired cognition, increased risk for cancer of the bladder, and HIV infections [6, 7].

There has been a surge in investment in the control of schistosomiasis as part of the Millennium Development Goals (MDGs) after 2000 and the Sustainable Development Goals (SDGs) from 2015 onwards [8, 9]. Reduction of disease burden is primarily achieved through preventive chemotherapy with the antischistosomal drug praziquantel [2, 10]. Treatment needs are commonly assessed with parasitologic methods [11–13]. In 2012, the World Health Organization (WHO) put forward a roadmap for elimination of schistosomiasis as a public health problem (i.e., prevalence of heavy infections under a threshold of 1%) and interruption of transmission in suitable settings by 2025 [14].

The WHO recommended diagnostic method for *S. haematobium* is urine filtration. A midday urine specimen is collected and, after vigorous shaking, 10 ml of urine are filtered and examined under a light microscope by experienced laboratory technicians [11, 15]. Alternative diagnostic techniques exist, which are often used in parallel on the same individual to increase diagnostic sensitivity. Blood in urine is a common symptom of urogenital schistosomiasis, although it is not fully specific to the disease [16]. As blood in urine is relatively easier to detect than *S. haematobium* eggs, three blood-based diagnostic tests are available; namely (i) a simple questionnaire regarding recent history of blood in the urine; (ii) inspection of a urine sample for visible blood; and (iii) a reagent strip for detection of microhematuria (detects visible as well as non-visible blood in urine) [17, 18]. Reagent strip for microhematuria allow for a semi-quantitative assessment of infection-intensity with four grades distinguishing the severity of an infection. Other diagnostic approaches include the detection of a specific antigen in urine and polymerase chain reaction (PCR)-based methods to detect genetic material in urine [19, 20].

In this study, we determined the infection intensity-dependent diagnostic accuracy of reagent strip to detect microhematuria and urine filtration for *S. haematobium* eggs. We considered repeated measurements obtained over consecutive days. Previous studies assessed the sensitivity and specificity of the aforementioned methods, but there is no study that models *S. haematobium* egg counts directly and takes into account day-to-day variation of infection intensity [15, 17, 21–24]. We extended our egg count model for individual-level data, previously developed for the analysis of Kato-Katz thick smears and a point-of-care circulating cathodic antigen (POC-CCA) urine cassette test for the diagnosis of *S. mansoni*. We developed a model to estimate ‘true’ prevalence and infection intensity-dependent sensitivity for urine filtration and reagent strip testing and determined the specificity of the latter diagnostic test [25, 26]. Additionaly, we employed a simulation to translate current WHO urine filtration intervention thresholds to microhematuria analogues based on reagent strip test results.

## Methods

### Ethics statement

All data included in this study have been published elsewhere [27]. Ethics approval and informed consent procedures are given in the aforementioned study from which the data have been extracted.

### Data

**Fig 1:**
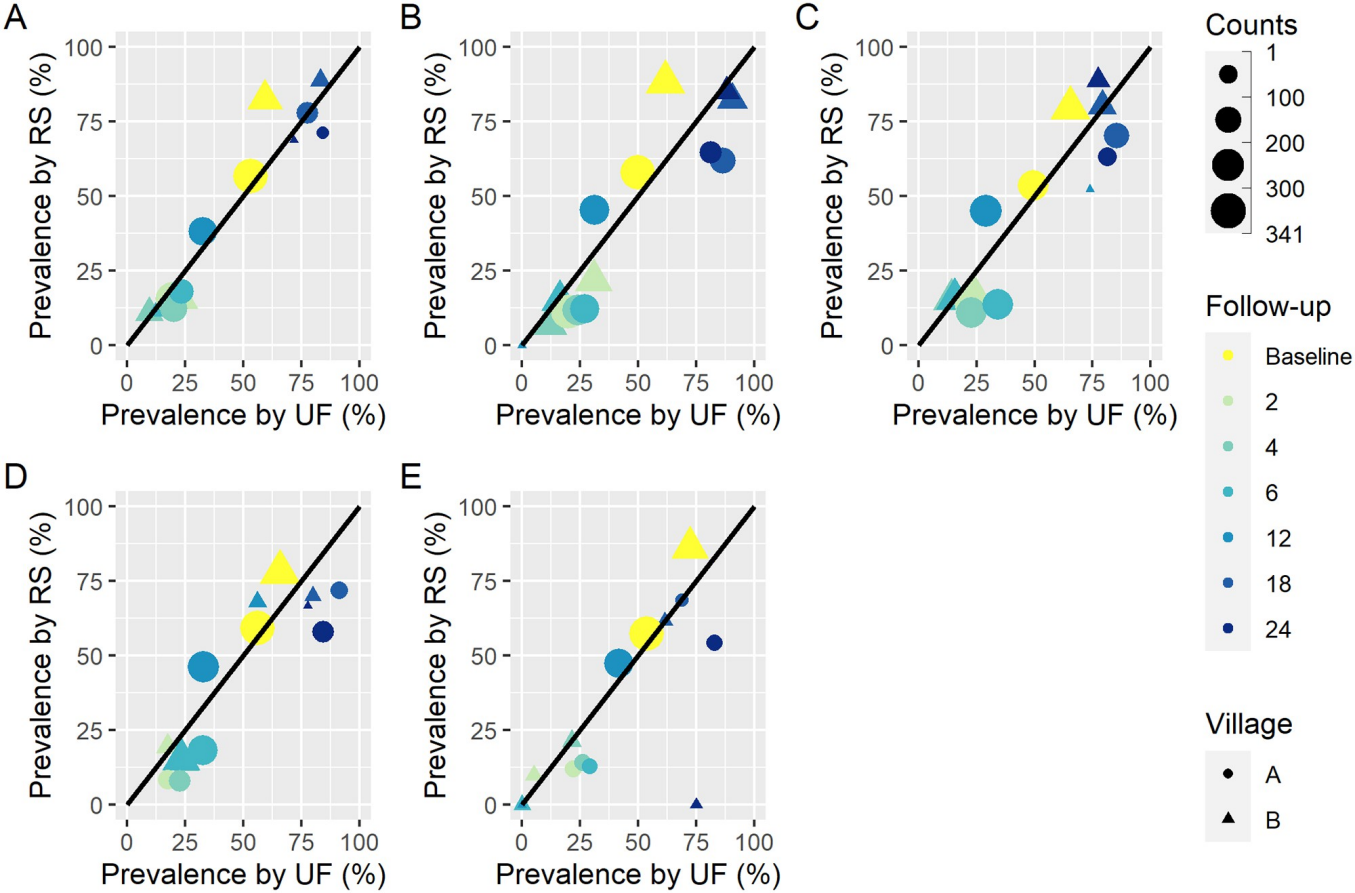
Comparison of prevalence by reagent strip (with trace negative) and urine filtration computed from raw data. The dots and triangles represent the prevalence at baseline (before treatment) and follow-up (at 2, 4, 6, 12, 18, and 24 months post-treatment) for each study site and ‘Counts’ show the number of individuals tested. **A** shows the prevalence computed from individuals with complete data at day 1 regarding reagent strip and urine filtration tests. **B, C, D**, and **E** show the same for days 2, 3, 4, and 5, respectively.

The analysis was carried out using a readily available dataset from a study conducted in two villages in Tanzania in 1993. The study involved 533 school-age children (7–18 years) and included a baseline and six follow-up surveys (at 2, 4, 6, 12, 18, and 24 months) after an initial treatment with praziquantel just after the baseline survey. At each survey, urine samples were collected and subjected to urine filtration and reagent strip testing (Boehringer Mannheim; Mannheim, Germany). Urine filtration was performed with samples of 10 ml of urine using Nucleopore membranes with 12 μl pore filters. Readings of the reagent strip were done semi-quantitatively. The results of the reagent strip used here consist of 0 (negative), trace T (<5 red blood cells (RBC)/μl of urine), 1+ (5–10 RBC/μl of urine), 2+ (∼50 RBC/μl of urine), and 3+ (∼250 RBC/μl of urine) [27]. For each survey, efforts were made to collect urine specimens over five consecutive days (between 10:00 and 14:00 hours). The aim of the study was to characterize the evolution of *S. haematobium* pathology after a single dose of praziquantel (40 mg/kg). However, 52 children were re-treated after 18 months (i.e., 6 months before the last follow-up) because they had heavy infections (≥ 50 eggs/10 ml urine), macrohematuria, or major lesions of the urinary tract. Summary measures of the data, stratified by village at the seven time points, are presented in Table 1 and a comparison of prevalence based on test results with reagent strip versus urine filtration for each day at the different time points is given in Fig 1.

**Table 1:** Observed prevalence and mean infection intensity by urine filtration and prevalence by microhematuria using reagent strip based on the first sample for all individuals and based on individuals with complete data, stratified by village (Tanzania survey, 1993 [27]).

Village	Survey	Urine filtration						Reagent strip					
		1 day all			1 day complete			1 day all			1 day complete		
		N	Positive (%)	μ^+^	N	Positive (%)	μ^+^	N	Positive T+^1^ (%)	Positive T-^2^ (%)	N	Positive T+^1^ (%)	Positive T-^2^ (%)
**A**	Baseline	310	56.5	203.4	152	56.6	187.5	310	59.4	53.2	152	59.2	53.3
	2 months	288	15.3	9.5	17	17.6	2.3	288	24.0	19.4	17	23.5	17.6
	4 months	204	12.3	37.8	20	0	0	204	23.0	20.1	20	20.0	20.0
	6 months	241	40.7	13.6	14	28.6	8.0	241	33.6	25.7	14	21.4	14.3
	12 months	219	37.9	31.1	91	35.2	24.6	219	47.5	32.4	91	49.5	31.9
	18 months	164	70.1	41.0	1	100	5.0	164	100	76.8	1	100	0
	24 months	122	57.4	23.5	0	-	-	122	100	75.4	0	-	-
**B**	Baseline	178	82.6	189.2	138	86.2	179.4	178	87.6	59.0	138	91.3	62.3
	2 months	163	15.3	14.4	13	7.7	1.0	163	42.3	23.3	13	15.4	7.7
	4 months	129	10.9	6.1	10	0	0	129	17.1	9.3	10	10.0	0
	6 months	172	23.8	10.4	14	0	0	172	25.0	16.3	14	0	0
	12 months	26	46.2	38.7	0	-	-	26	100	61.5	0	-	-
	18 months	103	79.6	153.2	1	100	6.0	103	100	79.6	1	100	100
	24 months	69	69.6	60.3	0	-	-	69	100	71.0	0	-	-

N is the number of individuals tested; μ^+^ is the mean number of eggs per 10 ml of urine in the positive individuals.

^1^ Trace results are regarded as positive.

^2^ Trace results are regarded as negative.

### *S. haematobium* egg count model

We assumed that the *S. haematobium* infection status and intensity of each individual was independent across follow-ups. For each individual, reagent strip (RS) data results were converted into 3 categorical variables with values T when the RS result was at least T (i.e., T/1+/2+/3+), 1 when the RS result was at least 1 (i.e., 1+/2+/3+), and 2 when the RS result was at least 2 (i.e., 2+/3+). The data consist of measurements over 5 consecutive days, and hence, each of the aforementioned variables were split into 5 binary variables corresponding to the *z* first days, *z* = 1 (i.e., first day of testing), 2 (i.e., first and second day), 3 (i.e., first 3 days), …, 5 (i.e., all 5 days) leading to 15 binary variables. Data from each cross-sectional time point and village were assumed to arise from a separate, independent population. As we have 7 cross-sectional time surveys corresponding to baseline and 6 follow-up surveys and 2 villages included in the study, we considered 14 independent populations. A joint Bayesian model was fitted separately for each binary RS variable with the corresponding urine filtration (UF) egg count. No diagnostic ‘gold’ standard was asummed. RS sensitivity for T,1,2 at *z* was related to infection intensity.

For each individual *i* in population *j*, *j* = 1, …, 14, let $Y_{jid}^{UF}$ be the observed egg counts from urine filtration on day *d*, *d* = 1, 2, …, 5. The results from a semi-quantitative reagent strip for microhematuria were coded into 15 binary variables: $Y_{ji}^{RS,T,z}$ describes all individuals with at least result trace, $Y_{ji}^{RS,1,z}$ with at least result 1+, and $Y_{ji}^{RS,2,z}$ 2+, respectively. The models were fitted separately for each of the 15 binary results $Y_{ji}^{RS,x,z}$, *x* = *T*, 1, 2, combined with all $Y_{jid}^{UF}$, where all individuals with both test results at a cross-sectional survey were included, with exception of a few individuals with multiple entries. We inferred on the sensitivity of repeated microhematuria measurements without having to model the correlation structure explicitly.

We assumed that each population consists of a proportion of infected individuals *π*_*j*_, where each individual had a disease status *D*_*ji*_ and infection intensity λ_*ji*_. Infected individuals with *D*_*ji*_ = 1 measurements on consecutive days were modeled as a negative binomial distribution with mean equal to the infection intensity λ_*ji*_ and dispersion parameter depending on it. The infection intesities were assumed to be distributed as a gamma distribution as follows:

$$\begin{array}{c} \begin{array}{cc} Y_{jid}^{UF} & ∼NB(\lambda_{ji},k_{ji}),\\ \text{log}(k_{ji}) & =k_{0}+\text{log}\left(\lambda_{ji}\right)k_{1},\text{and}\\ \lambda_{ji} & ∼Gamma(\mu_{j}^{+}\cdot \alpha_{j},\alpha_{j}),\text{where} \end{array} \end{array}$$ (1)

*k*_*ji*_ is the dispersion parameter of individual *i* in population *j* that depends on parameters *k*_0_ and *k*_1_, $\mu_{j}^{+}$ is the mean infection intensity of a positive individual in population *j*, and *α*_*j*_ is the aggregation parameter that describes the aggregation of infection intensities in population *j*. Individuals with infection status *D*_*ji*_ = 0 have observations $Y_{jid}^{UF}=0$, which is equivalent to 100% specificity. The sensitivity of urine filtration *s*_*jin*_ for individual *i* in population *j* after *n* days was calculated using the probability of repeated zero measurements under a negative binomial model, that is $s_{n}(\lambda )=1-{\left(\frac{k(\lambda )}{\lambda +k(\lambda )}\right)}^{n\cdot k(\lambda )}$, where $k\left(\lambda \right)=e^{k_{0}+\text{log}\left(\lambda \right)k_{1}}$.

Microhematuria measurements were modeled by a Bernoulli distribution,

$$\begin{array}{c} \begin{array}{cc} P(Y_{ji}^{RS,x,z}|D_{ji}) & =\{\begin{array}{cc} Be(s_{ji}^{RS,x,z})\; & ,\;\text{if}\;D_{ji}=1\\ Be(1-c^{RS,x,z})\; & ,\;\text{if}\;D_{ji}=0 \end{array}\\ \text{logit}(s_{ji}^{RS,x,z}/a_{3}^{x,z}) & =a_{0}^{x,z}+a_{1}^{x,z}\cdot \lambda_{ji}^{\text{log}(a_{2}^{x,z}+1)} \end{array} \end{array}$$ (2)

A parametric model was used to relate infection intensity with diagnostic sensitivity, where *s*^*RS*,*x*,*z*^ is the infection intensity-dependent sensitivity defined by four parameters. $a_{0}^{x,z}$ sets the sensitivity for infections approaching to zero intensity, $a_{1}^{x,z}$ describes the rate of increase with infection intensity, $a_{2}^{x,z}$ changes the shape of the curve, and $a_{3}^{x,z}$ limits the maximum sensitivity for very severe infections. *c*^*RS*,*x*,*z*^ is the specificity of the microhematuria reagent strip, that is the probability of no positive result for an uninfected individual where positive is defined as having at least once a value of *x* after *z* observations.

Models were also run with sensitivity and specificity parameters of the reagent strip stratified by sex. The model was formulated using a Bayesian paradigm. We chose a uniform prior for *π*_*j*_, a gamma distribution with mean 100 and standard deviation (SD) 100 for the mean infection intensity $\mu_{j}^{+}$, a gamma distribution with mean 1 and SD 1 for the population variation *α*_*j*_, a beta distribution with parameters 10 and 1 for *c*^*RS*^, and normal distributions with mean 0 and SD 2 for *k*_0_ and *k*_1_. For *a*_0_ a normal distribution with mean -1 and SD 1.5 was chosen, a gamma distribution with shape and scale parameters 5 and 30 for *a*_1_, a normal distribution with mean 10 and SD 10 for *a*_2_, and a beta distribution with parameters 10 and 1 for *a*_3_ to ensure a non-informative distribution of sensitivity curves in the relevant range of infection intensities. Priors for the sensitivity of the reagent strip were chosen using prior predictive checks to ensure non-informativity [28]. Other parameters have semi informative prior distributions over sensible parameter values. Inference was done using Markov chain Monte Carlo (MCMC) simulation. Model fit was carried out in Stan version 1.2.1335 (Stan Development Team; mc-stan.org), running 20 chains for 2,000 iterations of which the first 500 were discarded [29]. There were no divergent transitions, and convergence was assessed using the Gelman-Rubin diagnostics as well as visual inspection of chains [30].

### Simulation

To assess the relation between prevalence observed by microhematuria and urine filtration for one, two, three, four, and five days, we run extensive simulations of hypothetical populations in diverse transmission settings. We assumed that worms are negative binomially distributed in the population with worm aggregation parameter *w*^*agg*^ and that a proportion of 30% of the worms are female [31, 32]. The mean number of eggs per 10 ml of urine per female worm was selected from a publication of Truscott et al. who estimated it to be 5.2 [33]. For the parameters *a*_0_, *a*_1_, *a*_2_, *a*_3_, *k*_0_, and *k*_1_, 100 draws were taken directly from the posterior distribution and thereby correlation between parameters was incorporated in the simulation. The mean number of worms per individual in a population was varied in 40 equal steps on the log-scale from 1 to 200. We used *w*^*agg*^ according to a normal distribution with a mean of 0.2 and a SD of 0.03. For each hypothetical population, worm load for 6,000 individuals and corresponding urine filtration and reagent strip results were simulated for all five days.

## Results

### Descriptive data analysis

Prevalence by a single urine filtration ranged from 10.9% to 82.6%, while the cumulative prevalence over 5 consecutive days ranged from 20.9% to 98.7%. The mean infection intensity in the study population at the different time points ranged from 0.4 to 107 eggs/10 ml of urine, while the corresponding range in positive individuals was 1.8 to 116.6 eggs/10 ml. Reagent strip prevalence ranged from 17.1% to 100% when traces were considered positive and from 9.3% to 84.2% when traces were considered negative based on observations from a single day. Use of reagent strip over 5 consecutive days increased the observed prevalence from 33.9% to 100% when traces were considered positive and from 22.8% to 89.3% when traces were considered negative.

### Sensitivity of urine filtration

**Fig 2:**
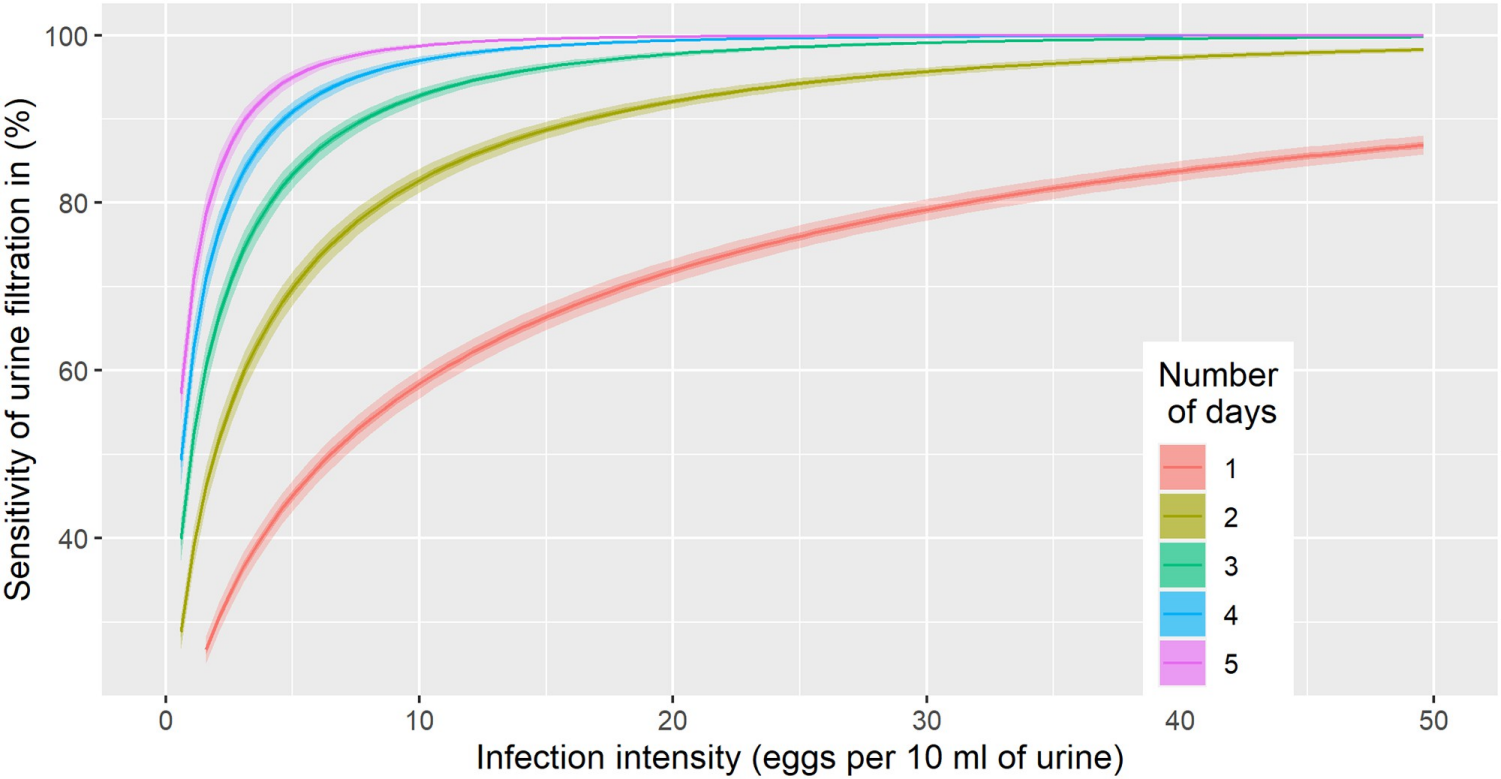
Sensitivity estimates of urine filtration (10 ml of urine) from a single to a total of five samples collected over consecutive days. Curves indicate posterior medians, while dark shaded areas provide a 50% and light shaded areas a 95% Bayesian credible interval.

Using the egg count model, we estimated the sensitivity of the urine filtration from a single sample to a total of five samples over consecutive days. The posterior medians for parameters *k*_0_ and *k*_1_, which define the relation between the sensitivity and mean infection intensity and were estimated at -2.3 and 0.4, respectively. The influence of the choice of *RS*, *x*, *z* on estimates of *k*_0_ and *k*_1_ was small (see S1 Text). The aggregation parameter of the negative binomial distribution increased from 0.15 to 0.48 for infection intensity increasing from 0 to 50 eggs/10 ml of urine, which corresponds to a reduction in overdispersion when the infection intensity increases. This is equivalent to a SD of 20 at a density of 10 eggs/10 ml of urine, 73 at a density of 50 eggs/10 ml of urine, and 3.3 at a density of 1 egg/10 ml of urine. The posterior distributions of *k*_0_ and *k*_1_ with the probability for repeated zeros under the negative binomial model enabled us to estimate the sensitivity of urine filtration. In Fig 2, we present the estimated sensitivity of urine filtration cumulatively for one to five days (i.e., single versus multiple tests) as a function of infection intensity.

A single urine filtration had a sensitivity of above 85% for heavy infections (≥50 eggs/10 ml of urine), while the sensitivity was below 50% at around 7 eggs/10 ml of urine. The sensitivity showed a considerable increase as a function of repeated urine filtration. For example, the sensitivity increased from 50% to 75% when comparing a single with a duplicate urine filtration at a low infection intensity of 7 eggs/10 ml of urine. After five days of urine filtration, an average infection with 1 egg/10 ml of urine showed a probability of around 60% to be detected.

### Sensitivity of reagent strip

The model was run for each interpretation of the reagent strip *RS*, *x*, *z* separately, where *x* is either *T* for trace, 1, or 2, and *z* the total number of samples from 1 to 5 (i.e., the test is positive when at least on one of the first *z* days a value of at least *x* in the semi-quantitative interpretation of the reagent strip is reported). The parameters *a*_0_, *a*_1_, *a*_2_, *a*_3_, and *c* fully define the diagnostic accuracy of each *RS*, *x*, *z*. The specificity *c* for each interpretation is summarized in Table 2. The specificity is estimated to basically 100% even after multiple days when only 2+ and 3+ test readings were considered positive while, when trace results were considered positive, the specificity of even a single sample is 97% and only 87% after five days.

**Table 2:** Specificity of each interpretation of the reagent strip *RS*, *x*, *z* defined as a result of at least *x* (*x* being T for trace, 1, or 2), on the first *z* days. Values are given between 0 and 1, where 1 corresponds to zero probability of a false-positive, values in brackets represent the 95% Bayesian credible interval.

Reading	RS,x,1	RS,x,2	RS,x,3	RS,x,4	RS,x,5
*x* = *T*	0.97 (0.92–1.00)	0.93 (0.86–0.99)	0.92 (0.85–0.99)	0.89 (0.82–0.98)	0.87 (0.80–0.96)
*x* = 1	0.99 (0.96–1.00)	0.99 (0.97–1.00)	0.99 (0.96–1.00)	0.98 (0.94–1.00)	0.97 (0.92–1.00)
*x* = 2	1.00 (0.98–1.00)	0.99 (0.98–1.00)	0.99 (0.97–1.00)	0.99 (0.97–1.00)	0.99 (0.96–1.00)

**Fig 3:**
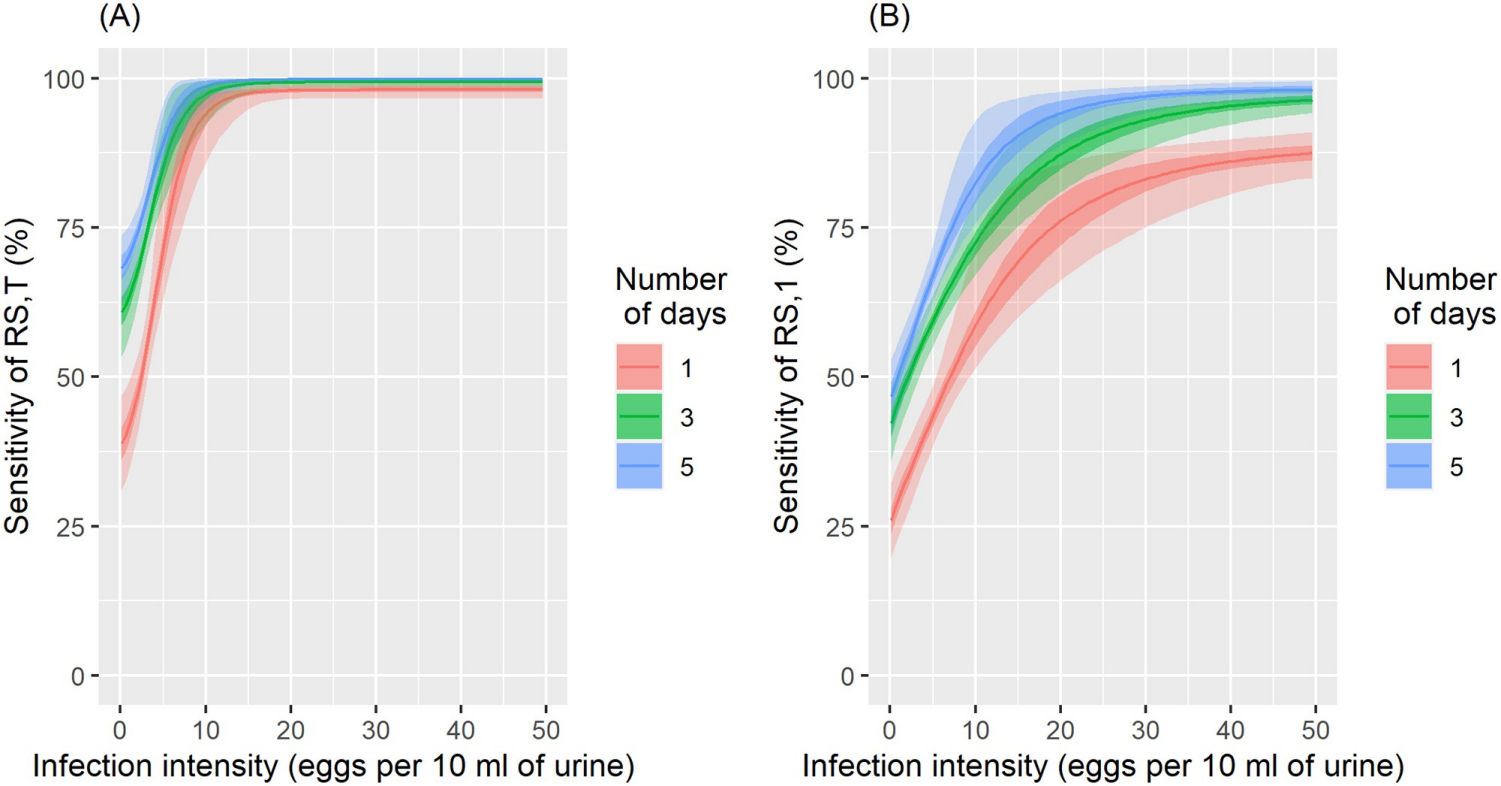
Sensitivity of a reagent strip (RS) in relation to infection intensity for measurements from a single to a total of 3 and 5 cumulative tests. Trace results were included in the positives (A) and trace results were included in the negatives (B). Dark shaded areas are the 50% and light shaded areas the 95% Bayesian credible interval.

The parameters *a*_0_, *a*_1_, *a*_2_, and *a*_3_ are reported for all 15 interpretations (S1 Text). Combined with Eq 2, these parameters estimate the infection intensity-dependent sensitivity of the reagent strip for microhematuria to diagnose *S. haematobium*. Fig 3A and 3B show the sensitivity of the reagent strip when traces were considered positive or negative, for cumulative results from one, three, and five days. When traces were considered positive, a single reagent strip detected basically all infections with an intensity above 15 eggs/10 ml of urine, and still 40% of infections with only 1 egg/10 ml of urine. Repeating the test over consecutive days increased the sensitivity, for example from 40% to 70% for very light infections after 5 consecutive days, but this increase was modest when compared to urine filtration.

Considering traces as negative resulted in higher specificity of 99% for a single reagent strip and still 97% after five samples. When traces were considered positive, the specificity after five samples was reduced to 87%, however, the sensitivity was higher compared to traces considered negative. A single reagent strip only has a 60% chance to detect an infection of 10 eggs/10 ml of urine and still less than 90% for heavy infections (≥50 eggs/10 ml of urine). Repeated sampling over 5 consecutive days increased the sensitivity up to 80% at 10 eggs/10 ml of urine compared to a sensitivity of almost 100% at the same intensity when traces were considered positive. The sensitivity of a single reagent strip when traces were considered negative is similar to a single urine filtration regardless of the level of infection intensity. Stratification by sex did not show any difference in parameter estimates that would indicate an important interaction, for example with menstruation.

**Fig 4:**
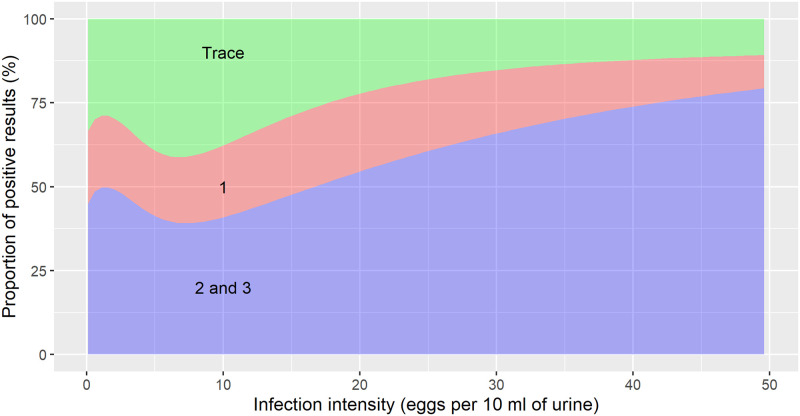
Proportion of semi-quantitative results of reagent strip for microhematuria in relation to *S. haematobium* infection intensity. The 2+ and 3+ readings were grouped together.

The semi-quantitative results of the reagent strip were closely correlated with the infection intensity of an individual. The proportion of trace, 1+, and >1+ results for infection intensities up to 50 eggs/10 ml of urine are shown in Fig 4. The non-monotonic behavior close to an infection intensity of zero is due to increased uncertainty in the sensitivity estimates (not shown in the plot, but visible in Fig 3A and 3B). At very low infection intensities, there was a considerable probability (∼40%) for readings of 2+ or 3+, while at 50 eggs/10 ml of urine almost 80% of tests showed a 2+ or 3+. Trace results, on the other hand, decreased from a proportion of about 30% to around 10% at 50 eggs/10 ml of urine, while the proportion of samples with reading 1+ remained relatively constant.

### Relation between the observed prevalence by urine filtration and reagent strip

**Fig 5:**
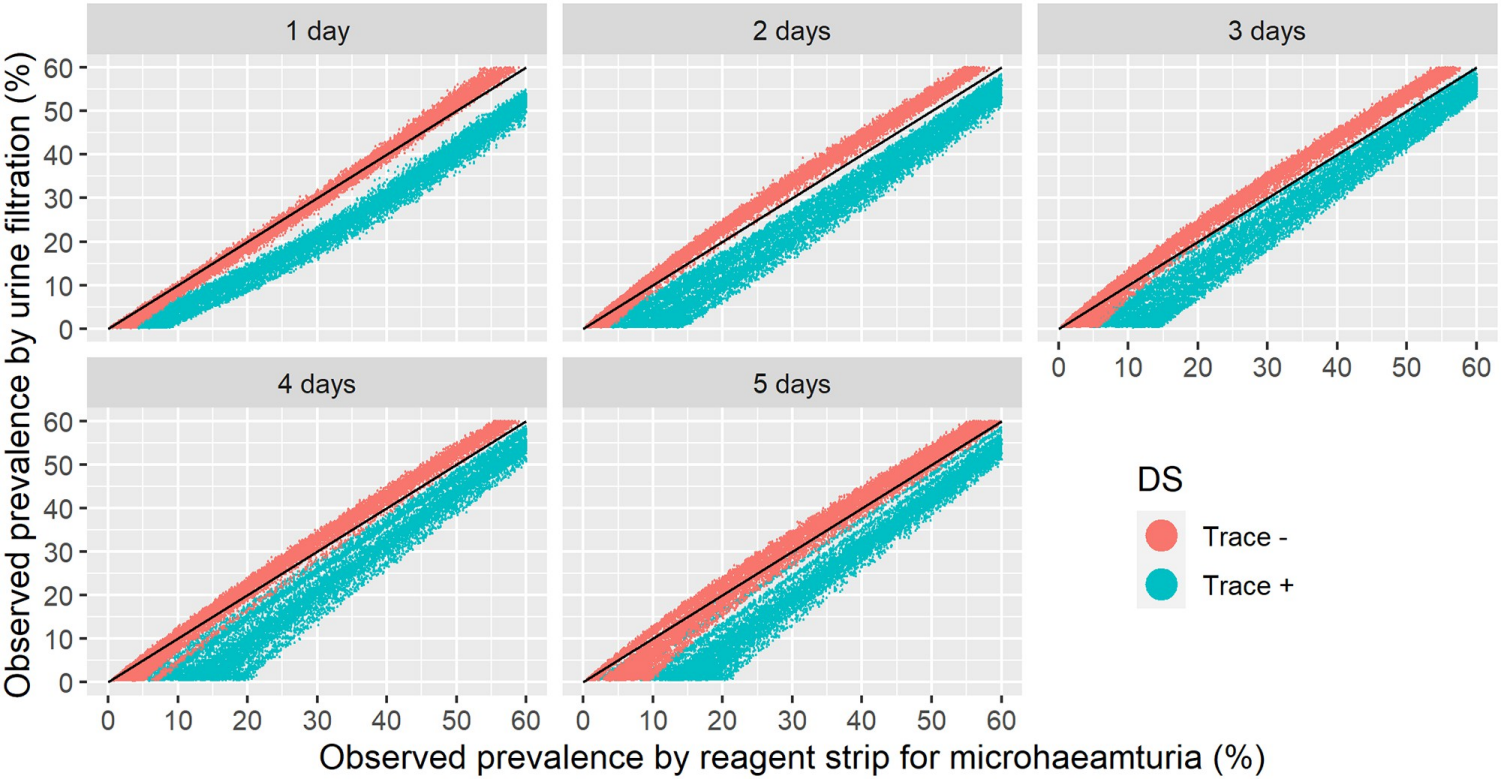
Simulated relation between observed prevalence of *S. haematobium* by urine filtration and by reagent strip based on one to five urine samples collected over consecutive days with traces considered either positive or negative. The black line indicates equivalence between urine filtration and reagent strip results.

We conducted extensive simulations of 3,000 hypothetical populations with 6,000 individuals each and generated observations of the two diagnostic tests over 5 consecutive days based on the individual-level results from the egg count model detailed above. Fig 5 depicts the relation between the observed prevalences by reagent strip and urine filtration for up to five samples collected over consecutive days. Traces were treated either as positive or negative. Measurements based on a single reagent strip reading showed equal or higher observed prevalence than urine filtration. When all five samples were considered, the prevalence was higher by urine filtration compared to reagent strip when traces were considered negative. For the sampling scheme recommended by WHO, i.e., single day sample, and reagent strip without trace results, the observed prevalence by both tests are similar, independent of the setting.

We translated WHO prevalence thresholds from urine filtration into microhematuria by taking all simulated populations with observed prevalence by urine filtration within a narrow interval of ±0.5% around the threshold and calculating the mean of the observed prevalence by reagent strip and the corresponding Bayesian credible intervals. Table 3 shows results for three thresholds of urine filtration, 10%, 25%, and 50%, and the reagent strip when traces were considered positive or negative, for sampling schemes based on a single up to five urine samples over consecutive days. Most relevant for evaluating treatment needs are results for sampling on a single day. When traces were considered negative, the prevalence thresholds for microhematuria were very close to urine filtration thresholds. Including traces in the positive required upwards adjustment of the thresholds, for example a 10% urine filtration corresponds to about 20% prevalence by reagent strip.

**Table 3:** Translation of prevalence thresholds from urine filtration (UF) into reagent strip (RS) for the diagnosis of *S. haematobium* for sampling schemes varying from one to five urine samples over consecutive days when traces were considered either positive or negative. All numerical values are percentages and the brackets contain 95% percentiles from the simulation.

RS	UF (%)	1 day	2 days	3 days	4 days	5 days
Traces +	10	17.6 (13.4–21.8)	16.5 (10.7–22.3)	19.2 (12.6–25.8)	19.4 (15.9–22.9)	21.4 (14.4–28.4)
	25	35.0 (31.6–38.5)	31.3 (25.7–36.8)	32.1 (27.8–36.5)	32.9 (29.2–36.6)	34.2 (28.1–40.3)
	50	57.7 (55.2–60.3)	54.8 (51.4–58.3)	54.2 (50.5–57.9)	55.6 (53.3–57.9)	56.2 (51.8–60.5)
Traces -	10	11.2 (9.0–13.3)	8.5 (7.2–9.8)	8.9 (6.9–11.0)	9.1 (6.9–11.3)	11.7 (7.7–15.6)
	25	25.7 (23.1–28.4)	21.5 (19.7–23.3)	21.4 (19.1–23.3)	22.7 (20.1–25.4)	24.0 (19.7–28.3)
	50	48.6 (46.6–50.6)	46.0 (44.4–47.6)	45.8 (449–47.6)	46.9 (44.5–49.3)	48.4 (45.5–51.3)

## Discussion

We determined the diagnostic accuracy of a reagent strip used for detection of microhematuria (a proxy for *S. haematobium* infection) from survey data where participants were subjected simultaneously to urine filtration and reagent strip testing, using a Bayesian egg count model. The study was conducted in the frame of a treatment intervention with a 24-month follow-up post-praziquantel intervention, thus characterized by different infection intensity. The semi-quantitative results of the reagent strip were incorporated into the modeling by determining the sensitivity and specificity profiles, when trace results of the reagent strip were considered either positive or negative, and when only the 2+ and 3+ readings were considered as positive. As the reagent strip results can be influenced by menstruation in females, stratification by sex was also done. Furthermore, we related the observed prevalence by the two tests and translated the urine filtration treatment prevalence thresholds recommended by WHO into thresholds by reagent strip. We analyzed the data cross-sectionally because the longitudinal nature captures the variation in the infection intensity, which is taken into account in the modeling by assuming that the sensitivity parameters of the two tests depend on the infection intensity.

For individual-level diagnosis, the reagent strip showed almost perfect sensitivity when infection intensity was above 15 eggs/10 ml of urine, while specificity was high (97%) for a single urine sample when traces were included in the positives. When traces were excluded, a much more similar profile to urine filtration was created across all infection intensities. A key difference between microhematuria and urine filtration is that results are more correlated across samples for microhematuria. This means that for urine filtration, an infection might be missed if tested only once, but captured by an additional urine filtration, whereas for microhematuria repeated tests will more likely reveal the same result leading to less improvement in sensitivity by repeating measurements. It is conceivable that the presence of blood in urine is less variable than the egg excretion by parasitic worms in the case where few worm-pairs are present as the eggs also may remain trapped in the walls of the urinary tract. The reduction in the overdispersion of the negative binomial distribution of egg outputs with increasing infection intensity supports arguments that in the presence of a larger number of worms, the day-to-day variations are low due to averaging. We did not observe a sex difference, which might be explained by the young age of the participants (7–18 years, 58% between 7 and 12 years) although a proportion of the females must have experienced menarche. Yet, the menstruation would have to have occurred at the same time as urine samples were collected and subjected to reagent strip testing to have an influence.

Point estimates of urine filtration and reagent strip sensitivity as well as specificity from other studies are difficult to compare to the results presented here because previous studies did not take into account the relation to infection intensity and they are averaged over the population. Hence, findings are setting specific. Urine filtration is considered the ‘gold’ standard for *S. haematobium* diagnosis [34]. However, results depend on the sampling scheme, as collection of multiple urine sample increases sensitivity. For example, a study conducted by Kosinski et al. (2011) reported a sensitivity of reagent strip using a single urine sample of about 50%, while the corresponding sensitivity for duplicate or triplicate reagent strip were 60% and 70%, respectively. For urine filtration, these estimates were 50%, 80%, and 100%, respectively [23]. Especially in low-endemic settings, a single urine filtration can lead to low sensitivity. Specificity of reagent strip testing decreases from 93% for single, to 88% for two, and 83% for three urine samples. These results are generally in agreement with our estimates, especially considering that those values over-estimate the diagnostic error because of the definition of urine filtration as ‘gold’ standard. Day-to-day variation in egg output is considerable, rendering a stable classification of infection intensity into severity classes difficult. Our study revealed that the semi-quantitative results derived from reagent strip testing are correlated with infection intensity, as determined by urine filtration. Variation in microhematuria seems to be lower, indicating that classification based on a reagent strip might be more stable and more insightful.

Our results were derived from the analysis of a study conducted in two villages in Tanzania in 1993 using a reagent strip produced by Boehringer Mannheim [27], a company that was bought and incorporated into Roche in 1997. It is unclear how representative the results are for other reagent strip tests currently on the market. For example, a study carried out in Kenya utilized a reagent strip called Hemastix (Ames, Bie and Bernsten; Copenhagen, Denmark). Mafe (1997) and Kahama et al. (1999), in studies carried out in Nigeria, also used Hemastix [17, 35]. More recently Kosinski et al. (2011) evaluated a semi-quantitative reagent strip called U-11 Urinalysis Reagent Strip (Mindray Co. Ltd.; Shenzhen, China). However, this test seems to have been on the market before; indeed, it has been evaluated by Mott et al. in 1985 [23, 36]. More data should be incorporated to study potential differences between reagent strips from different companies or from the same company over time. Some of the most recent data have been collected in the frame of large-scale preventive chemotherapy programs, which influence microhematuria following repeated praziquantel administration. Hence, this is an important confounder that should be included into future modeling studies.

On the population level, we see a clear relation between the observed prevalence by urine filtration and reagent strip. The size of the 3,000 simulated populations was fixed at 6,000 to be able to observe the influence of uncertainty in diagnostic accuracy, while limiting the influence of sampling error. Important assumptions in the simulation model were primarily the negative binomial distribution of worms in the population and the aggregation parameter that was considered to be independent of infection intensity. The latter may not hold because it has been assessed for hookworm infections, showing that the aggregation parameter increases with infection intensity [37]. In accordance with recommendations put forward by WHO and the Schistosomiasis Consortium for Operational Research and Evaluation (SCORE) for estimation of *S. haematobium* prevalence from a single day [14, 38], we recommend translating urine filtration thresholds of 10%, 25%, and 50% into 10%, 24%, and 48% when a single reagent strip is employed, considering traces as negative. Trace positive individuals should, however, also be treated with praziquantel, as it might indicate a very light infection with *S. haematobium* that might cause subtle morbidity [39, 40]. The reagent strip with traces considered negative serves as a convenient proxy to estimate prevalence almost equivalent to single-day urine filtration. If national control programs would make use of reagent strip instead of urine filtration, the costs could be reduced substantially and proceedings could be accelerated allowing more children to get tested.

Our study has several limitations that are offered for consideration. First, the data stem from a single type of reagent strip from a survey carried out in two villages of Tanzania almost 30 years ago with an age of participants ranging between 7 and 18 years. Importantly though, urine samples were obtained at multiple time points over the course of 24 months after a single oral dose of praziquantel. It is imperative to validate the results with additional data, for example using the Hemastix and U-11 reagent strips, particularly in settings where *S. haematobium* is close to elimination. Second, the simulation depends on the assumption of negative binomial distribution of worms in the population with a constant aggregation parameter, which is likely a good approximation at higher mean worm counts, but cannot be extrapolated to low prevalences below 10% observed urine filtration prevalence. This is reflected in our results making no recommendations for translation of lower thresholds. Third, it was not uncommon to have individuals where only one diagnostic test was performed on a specific sampling time point, and hence, observed prevalence by reagent strip and urine filtration at the same time point and village are not directly comparable.

Regarding the ethics of the analyzed study, the principal investigator emphasized that the children were treated when they were positive upon enrollment and then followed up for 24 months without treatment unless there were important symptoms. All infected children were treated at the end of the study, knowing that their potential or proven morbidity could be cleared with it. Based on previous experience, the study conductors could assume that within the 24 months, no serious morbidity that could not be reversed by treatment will occur, suggesting that such a study is ethically justifiable. The analyzed study was pivotal in defining the intervals possible for treatment and retreatment schemes that otherwise could not have been established for future actions.

### Conclusion

Reagent strip testing for microhematuria might be appropiate to test for *S. haematobium* infection, as it showed results with a high sensitivity and a high specificity. Indeed, at infection intensities above 15 eggs/10 ml of urine, sensitivity is close to 100%, while still maintaining a high specificity above 97%. When higher specificity is required, traces can be considered as negative to exclude the majority of false-positives, which lead to a sensitivity almost equal to a single sample urine filtration, therefore enabling direct translation of observed prevalence in the population. Further research is still warranted with additional data, particularly in settings where the prevalence of *S. haematobium* is low and infection intensity below 50 eggs/10 ml of urine.

## Supporting information

S1 Table Observed prevalence and mean infection intensity by urine filtration and prevalence by microhematuria using reagent strip on one and five cumulative days, stratified by village (Tanzania survey, 1993 [27]). (PDF)

S1 Text Tables with parameters describing the sensitivity and specificity of a reagent strip test for microhematuria including trace results (T), excluding trace results (1) and excluding trace and 1+ results (2) from one to five days of urine testing in a study pertaining to *S. haematobium* morbidity before and up to 24 months after praziquantel treatment. (PDF)

S1 Checklist STARD checklist. (DOCX)

S1 Data Individual level data. (CSV)
