# Supplementary material for: Infection intensity-dependent accuracy of reagent strip for the diagnosis of Schistosoma haematobium and estimation of treatment prevalence thresholds
Source: PLoS Negl Trop Dis. 2022 Apr 25;16(4):e0010332. doi: 10.1371/journal.pntd.0010332 (PMC9071146; doi:10.1371/journal.pntd.0010332)
Supplement: S1 Table — (PDF) [file pntd.0010332.s001.pdf]

S1 Table

Observed prevalence and mean infection intensity by urine filtration and prevalence by microhematuria using reagent strip on one and five cumulative days, stratified by village (Tanzania survey, 1993 [1]).

		**Urine filtration**						**Reagent strip**					
		**Day 1**		**5 days**				**Day 1**			**5 days**		
**Village**	**Survey**	N	Positive (%)	N	Positive (%)	μ	μ^+	N	Positive T+[1] (%)	Positive T-[2] (%)	N	Positive T+[1] (%)	Positive T-[2] (%)
**A**	Baseline	310	56.5	357	70.0	52.4	74.8	311	58.8	52.7	357	77.6	63.6
	2 months	288	15.3	328	22.3	0.8	3.5	287	24.0	19.5	328	38.4	27.7
	4 months	205	12.2	249	20.9	1.4	6.9	206	22.8	19.9	248	39.5	31.5
	6 months	241	40.7	244	59.0	5.5	9.4	172	32.0	23.3	244	50.4	39.3
	12 months	219	37.9	288	59.4	13.7	23.0	220	48.2	33.2	288	60.8	48.6
	18 months	202	56.9	237	69.2	22.9	33.1	141	100	77.3	187	100	89.3
	24 months	175	41.7	210	59.0	11.7	19.7	76	100	84.2	139	100	88.5
**B**	Baseline	178	82.6	207	91.8	107.0	116.6	179	86.6	58.7	207	93.2	81.6
	2 months	163	15.3	197	32.5	1.1	3.5	163	42.3	23.3	196	54.6	40.3
	4 months	129	10.9	171	24.6	0.4	1.8	129	17.1	9.3	171	33.9	22.8
	6 months	173	23.7	177	42.9	2.5	5.9	150	23.3	15.3	176	51.1	26.1
	12 months	67	23.9	76	44.7	4.8	10.8	0	-	-	32	96.9	65.6
	18 months	144	59.7	163	72.4	66.7	92.1	89	100	83.1	117	100	88.9
	24 months	113	44.2	151	50.3	36.8	73.0	68	98.5	70.6	85	98.8	81.2

N is the number of individuals tested; μ is the mean number of eggs per 10 ml of urine in the population after 5 tests on consecutive days; μ^+ is the mean number of eggs per 10 ml of urine in the positive individuals after 5 tests on consecutive days.

[1] Trace results are regarded as positive.

[2] Trace results are regarded as negative.

References

1. Hatz C, Vennervald BJ, Nkulila T, Vounatsou P, Kombe Y, Mayombana C, et al. Evolution of *Schistosoma haematobium*-related pathology over 24 months after treatment with praziquantel among school children in southeastern Tanzania. Am J Trop Med Hyg. 1998;59:775–781.
