# Supplementary material for: Infection intensity-dependent accuracy of reagent strip for the diagnosis of Schistosoma haematobium and estimation of treatment prevalence thresholds
Source: PLoS Negl Trop Dis. 2022 Apr 25;16(4):e0010332. doi: 10.1371/journal.pntd.0010332 (PMC9071146; doi:10.1371/journal.pntd.0010332)
Supplement: S1 Text — (PDF) [file pntd.0010332.s002.pdf]

# Supplementary Text S1

Parameters describing the sensitivity and specificity of a reagent strip test for microhaeamaturia including trace results (T) from one to 5 days of urine testing in a study pertaining to *S. haematobium* morbidity before and up to 2 years after praziquantel treatment.

| Number of tests | **1** | **2** | **3** | **4** | **5** |
|---|---|---|---|---|---|
| Reagent strip | | | | | |
| $c$ | 0.97 (0.92-1.00) | 0.93 (0.86-0.99) | 0.92 (0.85-0.99) | 0.89 (0.82-0.98) | 0.87 (0.80-0.96) |
| $a_0$ | -0.43 (-0.77--0.10 | 0.09 (-0.24-0.37) | 0.46 (0.14-0.73) | 0.67 (0.38-0.96) | 0.78 (0.49-1.04) |
| $a_1$ | 0.12 (0.04-0.27) | 0.10 (0.03-0.23) | 0.10 (0.03-0.24) | 0.09 (0.03-0.22) | 0.09 (0.03-0.21) |
| $a_2$ | 3.60 (1.90-8.29) | 3.88 (1.99-9.50) | 3.89 (1.94-9.74) | 4.02 (2.04-9.27) | 4.40 (2.18-10.69) |
| $a_3$ | 0.98 (0.97-0.99) | 0.99 (0.99-1.00) | 0.99 (0.99-1.00) | 1.00 (0.99-1.00) | 1.00 (0.99-1.00) |
| Urine filtration | | | | | |
| $k_0$ | -2.34 (-2.47--2.22) | -2.37 (-2.50--2.25) | -2.36 (-2.49--2.24) | -2.36 (-2.48--2.24) | -2.36 (-2.48--2.24) |
| $k_1$ | 0.38 (0.35-0.42) | 0.39 (0.36-0.42) | 0.39 (0.36-0.42) | 0.39 (0.36-0.42) | 0.39 (0.35-0.42) |

Parameters describing the sensitivity and specificity of a reagent strip for microhaeamaturia excluding trace results (1) from one to 5 days of urine testing in a study pertaining to *S. haematobium* morbidity before and up to 2 years after praziquantel treatment.

| Number of tests | **1** | **2** | **3** | **4** | **5** |
|---|---|---|---|---|---|
| Reagent strip | | | | | |
| $c$ | 0.99 (0.96-1.00) | 0.99 (0.97-1.00) | 0.99 (0.96-1.00) | 0.98 (0.94-1.00) | 0.97 (0.92-1.00) |
| $a_0$ | -0.93 (-1.31--0.60) | -0.59 (-0.89--0.31) | -0.29 (-0.59--0.04) | -0.16 (-0.47-0.10) | -0.12 (-0.43-0.14) |
| $a_1$ | 0.22 (0.08-0.47) | 0.21 (0.09-0.42) | 0.18 (0.07-0.37) | 0.17 (0.06-0.36) | 0.17 (0.06-0.36) |
| $a_2$ | 1.28 (0.73-2.83) | 1.15 (0.74-2.14) | 1.38 (0.86-2.69) | 1.61 (0.91-3.56) | 1.75 (0.97-3.94) |
| $a_3$ | 0.89 (0.84-0.95) | 0.97 (0.93-1.00) | 0.98 (0.95-1.00) | 0.98 (0.95-1.00) | 0.98 (0.96-1.00) |
| Urine filtration | | | | | |
| $k_0$ | -2.31 (-2.43--2.19) | -2.30 (-2.43--2.19) | -2.30 (-2.43--2.18) | -2.31 (-2.42--2.19) | -2.31 (-2.43--2.19) |
| $k_1$ | 0.38 (0.34-0.41) | 0.37 (0.34-0.41) | 0.37 (0.34-0.41) | 0.37 (0.34-0.41) | 0.37 (0.34-0.41) |

Parameters describing the sensitivity and specificity of a reagent strip for microhaeamaturia excluding trace and 1+ results (2) from one to 5 days of urine testing in a study pertaining to *S. haematobium* morbidity before and up to 2 years after praziquantel treatment.

| Number of tests | **1** | **2** | **3** | **4** | **5** |
|---|---|---|---|---|---|
| Reagent strip | | | | | |
| $c$ | 1.00 (0.98-1.00) | 0.99 (0.98-1.00) | 0.99 (0.97-1.00) | 0.99 (0.97-1.00) | 0.99 (0.96-1.00) |
| $a_0$ | -1.53 (-1.90--1.21) | -1.42 (-1.77--1.13) | -1.18 (-1.52--0.89) | -1.05 (-1.37--0.77) | -0.98 (-1.28--0.70) |
| $a_1$ | 0.28 (0.13-0.51) | 0.25 (0.11-0.46) | 0.22 (0.09-0.43) | 0.21 (0.09-0.41) | 0.20 (0.08-0.38) |
| $a_2$ | 0.87 (0.58-1.42) | 1.02 (0.70-1.62) | 1.14 (0.79-1.84) | 1.25 (0.85-2.02) | 1.40 (0.94-2.30) |
| $a_3$ | 0.93 (0.86-0.99) | 0.98 (0.94-1.00) | 0.99 (0.96-1.00) | 0.99 (0.96-1.00) | 0.99 (0.97-1.00) |
| Urine filtration | | | | | |
| $k_0$ | -2.31 (-2.43--2.18) | -2.31 (-2.43--2.19) | -2.30 (-2.42--2.18) | -2.30 (-2.41--2.17) | -2.30 (-2.42--2.18) |
| $k_1$ | 0.37 (0.34-0.41) | 0.37 (0.34-0.41) | 0.37 (0.34-0.40) | 0.37 (0.34-0.40) | 0.37 (0.34-0.40) |
