# Supplementary material for: Infection intensity-dependent accuracy of reagent strip for the diagnosis of Schistosoma haematobium and estimation of treatment prevalence thresholds
Source: PLoS Negl Trop Dis. 2022 Apr 25;16(4):e0010332. doi: 10.1371/journal.pntd.0010332 (PMC9071146; doi:10.1371/journal.pntd.0010332)
Supplement: S1 Checklist — (DOCX) [file pntd.0010332.s003.docx]

|  | **Section & Topic** | **No** | **Item** | **Reported on page #** |
| --- | --- | --- | --- | --- |
|  |  |  |  |  |
|  | **TITLE OR ABSTRACT** |  |  |  |
|  |  | **1** | Identification as a study of diagnostic accuracy using at least one measure of accuracy  (such as sensitivity, specificity, predictive values, or AUC)  **Text excerpt:** We developed a Bayesian model linking individual S. haematobium egg count data based on urine filtration to reagent strip binary test results available on multiple days and estimated the relation between infection intensity and sensitivity of reagent strip.  (Abstract: Methods) | p. 1 |
|  | **ABSTRACT** |  |  |  |
|  |  | **2** | Structured summary of study design, methods, results, and conclusions  (for specific guidance, see STARD for Abstracts)  Methods, Principal findings and conclusions from abstract. | p. 1 |
|  | **INTRODUCTION** |  |  |  |
|  |  | **3** | Scientific and clinical background, including the intended use and clinical role of the index test  **Text excerpts:** The WHO recommended diagnostic method for S. haematobium is urine filtration. Alternative diagnostic techniques exist, which are often used in parallel on the same individual to increase diagnostic sensitivity.  As blood in urine is relatively easier to detect than S. haematobium eggs, three blood-based diagnostic tests are available: (i) a simple questionnaire regarding recent history of visible blood in urine; (ii) inspection of a urine sample for visible blood; and (iii) a reagent strip for detection of microhematuria (detects visible as well as non-visible blood in urine) [1,2]. Reagent strip for microhematuria allow for a semi-quantitative assessment of infection-intensity with four grades distinguishing the severity of an infection. Other diagnostic approaches include the detection of a specific antigen in urine and polymerase chain reaction (PCR)-based methods to detect genetic material in urine [3,4]. | p. 2,3 |
|  |  | **4** | Study objectives and hypotheses  **Text excerpts:** In this study, we determined the infection intensity-dependent diagnostic accuracy of reagent strip to detect microhematuria and urine filtration for S. haematobium eggs. We considered repeated measurements obtained over consecutive days. Previous studies assessed the sensitivity and specificity of the aforementioned methods, but there is no  study that models S. haematobium egg counts directly and takes into account  day-to-day variation of infection intensity [5, 1, 6–9]. We extend our egg count  model for individual-level data, previously developed for the analysis of Kato-Katz thick  smears and a point-of-care circulating cathodic antigen (POC-CCA) cassette test for  diagnosis of S. mansoni. We developed a model to estimate ‘true’ prevalence and  infection intensity-dependent sensitivity for urine filtration and reagent strip testing and  determined the specificity of the latter diagnostic test [10, 11]. Finally, we employed a  simulation to translate current WHO urine filtration intervention thresholds to  microhematuria analogues based on reagent strip test results. | p. 3 |
|  | **METHODS** |  |  |  |
|  | *Study design* | **5** | Whether data collection was planned before the index test and reference standard  were performed (prospective study) or after (retrospective study)  This is a retrospective study.  **Text excerpt:** The analysis was carried out using a readily available dataset from a study conducted in two villages in Tanzania in 1993. | p. 3 |
|  | *Participants* | **6** | Eligibility criteria  **Text excerpts:** - The study involved 533 school-aged children (7-18 years) and included a baseline and six follow-up surveys (at 2,4,6, 12, 18 and 24 months) after an initial treatment with praziquantel just after the baseline survey.  At each survey, urine samples were collected and subjected to urine filtration and reagent strip testing (Boehringer Mannheim; Mannheim, Germany).  -For each survey efforts were made to collect urine specimens over five consecutive days (between 10:00 and 14:00 hours). The aim of the study was to characterize the evolution of S. haematobium pathology after a single dose of praziquantel. | p. 3 |
|  |  | **7** | On what basis potentially eligible participants were identified  (such as symptoms, results from previous tests, inclusion in registry)  **Text excerpts:** The analysis was carried out using a readily available dataset from a study conducted in two villages in Tanzania in 1993. The study involved 533 school-aged children (7-18 years) and included a baseline and six follow-up surveys (at 2,4,6, 12, 18 and 24  months) after an initial treatment with praziquantel just after the baseline survey. | p. 3 |
|  |  | **8** | Where and when potentially eligible participants were identified (setting, location and dates)  **Text excerpt:** The analysis was carried out using a readily available dataset from a study conducted in two villages in Tanzania in 1993. The study involved 533 school-aged children (7-18 years) and included a baseline and six follow-up surveys (at 2,4,6, 12, 18 and 24  months) after an initial treatment with praziquantel just after the baseline survey. For each survey, efforts were made to collect urine specimens over five consecutive days (between 10:00 and 14:00 hours). | p. 3 |
|  |  | **9** | Whether participants formed a consecutive, random or convenience series  **Text excerpt:** The analysis was carried out using a readily available dataset from a study conducted in two villages in Tanzania in 1993. The study involved 533 school-aged children (7-18 years) and included a baseline and six follow-up surveys (at 2,4,6, 12, 18 and 24  months) after an initial treatment with praziquantel just after the baseline survey. | p. 3 |
|  | *Test methods* | **10a** | Index test, in sufficient detail to allow replication  **Text excerpts:** The study involved 533 school-age children (7-18 years) and included a baseline and six follow-up surveys (at 2, 4, 6, 12, 18, and 24 months) after an initial treatment with praziquantel just after the baseline survey. At each survey, urine samples were collected and subjected to urine filtration and reagent strip testing (Boehringer Mannheim; Mannheim, Germany). Readings of the reagent strip were done semi-quantitatively with values corresponding to 0 (negative), trace T (< 5 red blood cells (RBC)/µl of urine), 1+ (5-10 RBC/µl of urine), 2+ (~50 RBC/µl of urine), and 3+ (~250 RBC/µl of urine) [12]. | p. 3 |
|  |  | **10b** | Reference standard, in sufficient detail to allow replication  **Text excerpts:** The study involved 533 school-age children (7-18 years) and included a baseline and six follow-up surveys (at 2, 4, 6, 12, 18, and 24 months) after an initial treatment with praziquantel just after the baseline survey. At each survey, urine samples were collected and subjected to urine filtration and reagent strip testing (Boehringer Mannheim; Mannheim, Germany). Urine filtration was performed with samples of 10 ml of urine using Nucleopore membranes with 12 µl pore filters. For each survey, efforts were made to collect urine specimens over five consecutive days (between 10:00 and 14:00 hours). | p. 3 |
|  |  | **11** | Rationale for choosing the reference standard (if alternatives exist)  **Text excerpt:** The analysis was carried out using a readily available dataset from a study conducted in two villages in Tanzania in 1993. At each survey, urine samples were collected and subjected to urine filtration and reagent strip testing (Boehringer Mannheim; Mannheim, Germany). | p. 3 |
|  |  | **12a** | Definition of and rationale for test positivity cut-offs or result categories  of the index test, distinguishing pre-specified from exploratory  **Text excerpt:** Readings of the reagent strip were done semi-quantitatively with values corresponding to 0 (negative), trace (< 5 red blood cells (RBC)/µl of urine), 1+ (5 − 10 RBC/µl of urine), 2+ (∼ 50 RBC/µl of urine), and 3+ (∼ 250 RBC/µl of urine) [12]. For each individual, reagent strip (RS) data results were converted into 3 categorical variables with values T when the RS result was at least T (i.e. T/1+/2+/3+), 1 when the RS result was at least 1 (i.e. 1+/2+/3+) and 2 when the RS result was at least 2 (i.e. 2+/3+). | p. 3 |
|  |  | **12b** | Definition of and rationale for test positivity cut-offs or result categories  of the reference standard, distinguishing pre-specified from exploratory  **Text excerpt:** Urine filtration was performed with samples of 10 ml of urine using Nucleopore membranes with 12 µl pore filters. For each survey, efforts were made to collect urine specimens over five consecutive days (between 10:00 and 14:00 hours). | p. 3 |
|  |  | **13a** | Whether clinical information and reference standard results were available  to the performers/readers of the index test  **Text excerpt:** The analysis was carried out using a readily available dataset from a study conducted in two villages in Tanzania in 1993. At each survey, urine samples were collected and subjected to urine filtration and reagent strip testing (Boehringer Mannheim; Mannheim, Germany). | p. 3 |
|  |  | **13b** | Whether clinical information and index test results were available  to the assessors of the reference standard  **Text excerpt:** The analysis was carried out using a readily available dataset from a study conducted in two villages in Tanzania in 1993. At each survey, urine samples were collected and subjected to urine filtration and reagent strip testing (Boehringer Mannheim; Mannheim, Germany). | p. 3 |
|  | *Analysis* | **14** | Methods for estimating or comparing measures of diagnostic accuracy  **Text excerpt:** For each individual i in population j, j = 1, . . . , 14 let Yjid be the observed egg  counts from urine filtration on day d, d = 1, 2, . . . , 5. The results from a  semi-quantitative reagent strip for microhematuria were coded  into 15 binary variables: YjiRS,T,z describes all individuals with at least result trace,  YjiRS,1,z with at least result 1+ and YjiRS,2,z 2+, repectively. z denotes the first days  (z = 1, . . . , 5), i.e. z = 1 refers to the test result of the first day, z = 2 refers to the  results of the first two days and so forth. The models were fitted separately for each of  UF, where all the 15 binary results YjiRS,x,z , x = T, 1, 2, combined with all Yjid  observations were included. We inferred on the sensitivity of  repeated microhematuria measurements without having to model the correlation  structure explicitly. | p. 4 |
|  |  | **15** | How indeterminate index test or reference standard results were handled  **Text excerpt:** All data included in this study have been published elsewhere [12]. Ethics approval and informed consent procedures are given in the aforementioned study from which the data have been extracted. | p. 3 |
|  |  | **16** | How missing data on the index test and reference standard were handled  **Text excerpts:** -The models were fitted separately for each of the 15 binary results Y RS;x;zji , x = T; 1; 2, combined with all Y UFjid , where all individuals with both test results at a cross-sectional survey were included, with exception of a few individuals with multiple entries. | p. 4 |
|  |  | **17** | Any analyses of variability in diagnostic accuracy, distinguishing pre-specified from exploratory  To assess the relation between prevalence observed by microhematuria and urine  filtration for one, two, three, four, and five days, we run extensive simulations of hypothetical populations in diverse transmission settings. We assumed that worms are negative binomially distributed in the population with worm aggregation parameter wagg and that a proportion of 30% of the worms are female [13,14]. The mean number of eggs per 10 ml of urine per  female worm was selected from a publication of Truscott et al. who estimated it to be 5.2 [15]. For the parameters a0 , a1 , a2 , a3 , k0 , and k1 100 draws  were taken directly from the posterior distribution and thereby correlation between  parameters was incorporated in the simulation. The mean number of worms per  individual in a population was varied in 30 equal steps on the log-scale from 1 to 400.  wagg according to a normal distribution with a mean of 0.2 and a SD of 0.03. For each  population worm load for 5000 individuals and corresponding urine filtration and  reagent strip results were simulated for all five days. | p. 5 |
|  |  | **18** | Intended sample size and how it was determined  **Text excerpt:** All data included in this study have been published elsewhere [12]. Ethics approval and informed consent procedures are given in the aforementioned study from which the data have been extracted. | p. 3 |
|  | **RESULTS** |  |  |  |
|  | *Participants* | **19** | Flow of participants, using a diagram  **Text excerpt:** All data included in this study have been published elsewhere [12]. Ethics approval and informed consent procedures are given in the aforementioned study from which the data have been extracted. | p. 3 |
|  |  | **20** | Baseline demographic and clinical characteristics of participants  **Text excerpt:** All data included in this study have been published elsewhere [12]. Ethics approval and informed consent procedures are given in the aforementioned study from which the data have been extracted. | p. 3 |
|  |  | **21a** | Distribution of severity of disease in those with the target condition  **Text excerpt:** All data included in this study have been published elsewhere [12]. Ethics approval and informed consent procedures are given in the aforementioned study from which the data have been extracted. | p. 3 |
|  |  | **21b** | Distribution of alternative diagnoses in those without the target condition  **Text excerpt:** All data included in this study have been published elsewhere [12]. Ethics approval and informed consent procedures are given in the aforementioned study from which the data have been extracted. | p. 3 |
|  |  | **22** | Time interval and any clinical interventions between index test and reference standard  **Text excerpt:** The study involved 533 school-aged children (7-18 years)  and included a baseline and six follow-up surveys (at 2, 4, 6, 12, 18 and 24  months) after an initial treatment with praziquantel just after the survey. | p. 3 |
|  | *Test results* | **23** | Cross tabulation of the index test results (or their distribution)  by the results of the reference standard  Figure 1, Table 1, Table 2 | p. 4, 6, 7 |
|  |  | **24** | Estimates of diagnostic accuracy and their precision (such as 95% confidence intervals)  Table 1, 2 and 3  **Text excerpts:** -A single urine filtration had a sensitivity of above 85% for heavy infections (≥ 50 eggs per 10 ml of urine) while the sensitivity was below 50% at around 7 eggs per 10 ml  of urine. The sensitivity showed a substantial increase as a function of repeated urine  filtration. For example the sensitivity increased from 50% to 75% when comparing a  single with a double urine filtration at a low infection intensity of 7 eggs per 10 ml of  urine. After five days of urine filtration, an average infection with a single worm-pair at  1 egg per 10 ml of urine showed a probability of around 60% to be detected.  - Considering traces as negative resulted in higher specificity of 99% for a single  reagent strip and still 95% after five samples. When traces were considered positive, the  specificity after 5 samples was reduced to 85%, however, the sensitivity was higher  compared to traces considered negative. A single reagent strip has only a 60% chance to  detect an infection of 10 eggs per 10 ml of urine and still less than 90% for heavy  infections (≥ 50 eggs per 10 ml of urine). Repeated sampling over five consecutive days  increased the sensitivity up to 80% at 10 eggs per 10 ml of urine compared to a  sensitivity of almost 100% at the same intensity when traces were considered positive.  The sensitivity of a single reagent strip when traces were considered negative is  similar to a single urine filtration regardless of the level of infection intensity.  Stratification by sex did not show any difference in parameter estimates that would  indicate an important interaction for example with menstruation.  - The semi-quantitative results of the reagent strip were closely correlated with the  infection intensity of an individual. The proportion of trace, 1 and > 1 results for  infection intensities up to 50 eggs per 10 ml of urine are shown in Fig 4. The  non-monotonic behavior close to an infection intensity of zero is due to increased  uncertainty in the sensitivity estimates (not shown in the plot, but visible in Figs 3a  and 3b). At very low infection intensities, there was a considerable probability (∼ 40%)  for readings of 2 or 3, while at 50 eggs per 10 ml of urine almost 80% of tests showed a  2 or 3. Trace results, on the other hand, decreased from a proportion of about 30% to  less than 10% at 50 eggs per 10 ml of urine while the proportion of samples with  reading 1 remained relatively constant.  - We translated the WHO prevalence thresholds from urine filtration into  microhematuria by taking all simulated populations with observed prevalence by urine  filtration within a narrow interval of ±0.5% around the threshold and calculating the  mean of the observed prevalence by reagent strip and the corresponding Bayesian  credible intervals. Table 3 shows results for three thresholds of urine filtration,  10%, 25%, and 50%, and the reagent strip when traces were considered positive or  negative, for sampling schemes based on a single up to five urine samples over  consecutive days. Most relevant for evaluating treatment needs are results for sampling  on a single day. When traces were considered negative, the prevalence thresholds for  microhematuria were very close to urine filtration thresholds. Including traces in the  positive required upwards adjustment of the thresholds, for example a 10% urine  filtration corresponds to about 20% prevalence by reagent strip. | p. 6-9 |
|  |  | **25** | Any adverse events from performing the index test or the reference standard  **Text excerpt:** All data included in this study have been published elsewhere [12]. Ethics approval and informed consent procedures are given in the aforementioned study from which the data have been extracted. | p. 3 |
|  | **DISCUSSION** |  |  |  |
|  |  | **26** | Study limitations, including sources of potential bias, statistical uncertainty, and generalisability  **Text excerpt:** Our study has several limitations that are offered for consideration. First, the data stem from a single type of reagent strip from a survey carried out in only two villages of Tanzania almost 30 years ago with an age of participants ranging between 7 and 18 years. Importantly though, urine samples were obtained at multiple time points over the course of 24 months after a single oral dose of praziquantel. It is imperative to validate the results with additional data, for example using the Hemastix and U-11 reagent strip, particularly in settings where S. haematobium is close to elimination. Second, the simulation depends on the  assumption of negative binomial distribution of worms in the population with a  constant aggregation parameter, which is likely a good approximation at higher mean  worm counts but cannot be extrapolated to low prevalences below 10% observed urine  filtration prevalence. This is reflected in our results making no recommendations for  translation of lower thresholds. Third, it was not uncommon to have individuals  where only one diagnostic test was performed on a specific sampling time point, and  hence, observed prevalence by reagent strip and urine filtration at the same time point, and village are not directly comparable. | p. 11 |
|  |  | **27** | Implications for practice, including the intended use and clinical role of the index test  **Text excerpt:** In accordance with recommendations put forward by WHO and the Schistosomiasis Consortium for Operational Research and Evaluation (SCORE) for estimation of S. haematobium prevalence from a single day [16,17], we recommend translating urine filtration thresholds of 10%, 25% and 50% into 12%, 26% and 50% when a single reagent strip is employed, considering traces as negative. Trace positive individuals should, however, also be treated with praziquantel, as it might indicate a very light infection with S. haematobium that might cause subtle morbidity [18,19]. The reagent strip with traces considered negative serves as a convenient proxy to estimate prevalence almost equivalent to single-day urine filtration. | p. 10-11 |
|  | **OTHER INFORMATION** |  |  |  |
|  |  | **28** | Registration number and name of registry  **Text excerpt:** All data included in this study have been published elsewhere [12]. Ethics approval and informed consent procedures are given in the aforementioned study from which the data have been extracted. | p. 3 |
|  |  | **29** | Where the full study protocol can be accessed  **Text excerpt:** All data included in this study have been published elsewhere [12]. Ethics approval and informed consent procedures are given in the aforementioned study from which the data have been extracted. | p. 3 |
|  |  | **30** | Sources of funding and other support; role of funders  **Text excerpt:** This study received financial support from the European Research Council  (ERC-2012-AdG-323180) and the Schistosomiasis Consortium for Operational Research  and Evaluation (SCORE). The funders had no role in study design, data collection and  analysis, decision to publish, or preparation of the manuscript. | p. 12 |
|  |  |  |  |  |

References

1. Mafe MA. The diagnostic potential of three indirect tests for urinary schistosomiasis in Nigeria. Acta Trop. 1997;68:277-284.
2. Lengeler C, Makwala J, Ngimbi D, Utzinger J. Simple school questionnaire can map both *Schistosoma mansoni* and *Schistosoma haematobium* in the Democratic Republic of Congo.

Acta Trop. 2000;74:77-87.

1. Knopp S, Corstjens PLAM, Koukounari A, Cercamondi CI, Ame SM, Ali SM, et al. Sensitivity and specificity of a urine circulating anodic antigen test for the diagnosis of *Schistosoma haematobium* in low endemic settings. PLoS Negl Trop Dis. 2015;9:e0003752.
2. Shiff C. Accurate diagnostics for schistosomiasis: a new role for PCR. Rep Parasitol. 2015;4:23--29.
3. Knopp S, Ame SM, Hattendorf J, Ali SM, Khamis IS, Bakar F, et al. Urogenital schistosomiasis elimination in Zanzibar: accuracy of urine filtration and haematuria reagent strips for diagnosing light intensity *Schistosoma haematobium* infections. Parasit Vectors. 2018;11:552.
4. Obeng BB, Aryeetey YA, de Dood CJ, Amoah AS, Larbi IA, Deelder AM, et al. Application of a circulating-cathodic-antigen ({CCA}) strip test and real-time {PCR}, in comparison with microscopy, for the detection of *Schistosoma haematobium* in urine samples from Ghana. Ann Trop Med Parasitol. 2008;102:625--633.
5. Midzi N, Butterworth AE, Mduluza T, Munyati SM, Deelder AM, van Dam G. Use of circulating cathodic antigen strips for the diagnosis of urinary schistosomiasis. Trans R Soc Trop Med Hyg. 2009;103:45--51.
6. Kosinski KC, Bosompem K, Stadecker MJ, Wagner AD, Plummer J, Durant JL, et al. Diagnostic accuracy of urine filtration and dipstick tests for *Schistosoma haematobium* infection in a lightly infected population of Ghanaian schoolchildren. Acta Trop. 2011;118:123--127.
7. Stete K, Krauth SJ, Coulibaly JT, Knopp S, Hattendorf J, Müller I, et al. Dynamics of *Schistosoma haematobium* egg output and associated infection parameters following treatment with praziquantel in school-aged children. Parasit Vectors. 2012;5:298.
8. Bärenbold O, Raso G, Coulibaly JT, N'Goran EK, Utzinger J, Vounatsou P. Estimating sensitivity of the {K}ato-{K}atz technique for the diagnosis of *Schistosoma mansoni* and hookworm in relation to infection intensity. PLoS Negl Trop Dis. 2017;11:e0005953.
9. Bärenbold O, Garba A, Colley DG, Fleming FM, Haggag AA, Ramzy RMR, et al. Translating preventive chemotherapy prevalence thresholds for *Schistosoma mansoni* from the Kato-Katz technique into the point-of-care circulating cathodic antigen diagnostic test. PLoS Negl Trop Dis. 2018;12:e0006941.
10. Hatz C, Vennervald BJ, Nkulila T, Vounatsou P, Kombe Y, Mayombana C, et al. Evolution of *Schistosoma haematobium-*related pathology over 24 months after treatment with praziquantel among school children in southeastern Tanzania. Am J Trop Med Hyg. 1998;59:775--781.
11. Anderson RM. The population dynamics and epidemiology of intestinal nematode infections. Trans R Soc Trop Med Hyg. 1986;80:686--696.
12. May RM, Woolhouse MEJ. Biased sex ratios and parasite mating probabilities. Parasitology. 1993;107:287--295.
13. Truscott JE, Gurarie D, Alsallaq R, Toor J, Yoon N, Farrell SH, et al. A comparison of two mathematical models of the impact of mass drug administration on the transmission and control of schistosomiasis. Epidemics. 2017;18:29-37.
14. WHO. Schistosomiasis: progress report 2001--2011 and strategic plan 2012--2020. Geneva: World Health Organization; 2013.
15. King CH, Bertsch D, Andrade GN, Burnim M, Ezeamama AE, Binder S, et al. The Schistosomiasis Consortium for Operational Research and Evaluation rapid answers project: systematic reviews and meta-analysis to provide policy recommendations based on available evidence. Am J Trop Med Hyg. 2020;103(1 Suppl):92-96.
16. King CH, Sturrock RF, Kariuki HC, Hamburger J. Transmission control for schistosomiasis - why it matters now. Trends Parasitol. 2006;22:575-82.
17. King CH, Bertsch D. Meta-analysis of urine heme dipstick diagnosis of *Schistosoma haematobium* infection, including low-prevalence and previously-treated populations. PLoS Negl Trop Dis. 2013;7:e2431.
